# Facilitating and Hindering Factors of Health Help-Seeking Behavior in Patients with Chronic Diseases: A Qualitative Study

**DOI:** 10.3390/healthcare13172164

**Published:** 2025-08-29

**Authors:** Linlin Su, Xiaochen Lv, Xiao Yang, Xiaofan Wang, Lixia Qu, Chunhui Zhang

**Affiliations:** School of Nursing and Health, Zhengzhou University, Zhengzhou 450001, China; sulinlin2023@gs.zzu.edu.cn (L.S.); xiaochenlv2023@163.com (X.L.); zzu2024yx@163.com (X.Y.); wangxf202409@163.com (X.W.)

**Keywords:** chronic diseases, noncommunicable diseases, help-seeking behavior, health services accessibility, qualitative research

## Abstract

(1) **Background**: Help-seeking behavior is a key way to maintain health and seek effective treatment, and it also helps to improve patients’ self-management ability. This study aimed to investigate the facilitating and hindering factors of help-seeking behaviors among patients with chronic diseases concerning their health issues. (2) **Methods**: Based on the Capability, Opportunity, and Motivation-Behavior (COM-B) model, 18 patients with chronic diseases in a tertiary hospital in Zhengzhou City, Henan Province, were selected for semi-structured in-depth interviews between July and November 2024 using a descriptive qualitative research approach. The collected data were analyzed using directed content analysis. (3) **Results**: A total of 18 interviews were conducted, and two themes and six sub-themes were extracted. The factors that promote health help-seeking behavior in patients with chronic diseases include ability (self-health monitoring ability, sufficient communication preparation ability), opportunity (health support in social bonds, effective support of medical staff), and motivation (good illness identity, past successful experience of health seeking help). Barriers include ability (symptom attribution bias, difficulty in identifying health information), opportunity (heavier financial burden, poor sense of gain in interactions), and motivation (fear and avoidance, stigma of illness). (4) **Conclusions**: There are some hindering factors and obvious contributing factors regarding health help-seeking behavior among patients with chronic diseases. Medical staff should prioritize guiding patients to seek help for health problems. The COM-B model can be applied to develop targeted intervention strategies for improving help-seeking behavior. This approach is beneficial for enhancing patients’ health management capabilities by promoting proactive health help-seeking practices.

## 1. Introduction

Chronic disease is an important issue in global public health [1]. According to statistics [2], the prevalence of chronic diseases among middle-aged and elderly people over 50 years of age in China is as high as 61.9%, and chronic diseases have become a major problem affecting the health and quality of life of middle-aged and elderly groups [3]. Studies have shown that timely health help-seeking is a key way to maintain health and seek effective treatment [4], and that improving health help-seeking behavior also helps to improve patients’ self-management ability, which in turn improves their quality of life [5]. Help-seeking behaviors in patients with chronic diseases can be understood as a multi-stage process of health problem-focused, planned behaviors involving interpersonal interactions with healthcare professionals [6].

The outline of the “Healthy China 2030” plan clearly emphasizes promoting health management for the entire population, focusing on enhancing health literacy and everyone taking responsibility for their own health [7]. Taking the initiative to seek help for health issues is, to a large extent, a manifestation of being responsible for one’s own health. It reflects the importance attached to one’s own health and an attitude of positive management. Help-seeking behavior is also crucial for improving healthcare in an aging society [8]. Furthermore, some studies have pointed out that patients who are diabetic ignore foot symptoms and fail to seek medical help in time, which results in irreversible consequences such as amputation [9].

However, the current status of health help-seeking behavior in patients with chronic diseases is concerning. It is primarily characterized by delayed treatment initiation, non-adherence to prescribed treatments, an increased risk of complications, and underutilization of available health services [10,11,12,13]. Elderly patients with chronic diseases often fail to fully assess their own health conditions. They are not clear about whether they need help, what problems to seek help for, when to seek help, and to whom to turn for help. Often, they do not seek help but choose to wait for the symptoms to disappear [14]. A survey of 2073 rural elderly people in China shows that 90% of them do not pay attention to self-care, 90% only start to care about their health after falling ill, and over 76% only seek medical treatment when they are seriously ill [15]. Therefore, paying attention to the health assistance behaviors of patients with chronic diseases and exploring the promoting and hindering factors of their assistance behaviors have important theoretical and practical significance.

Currently, research on the influencing factors of help-seeking behavior primarily focuses on two aspects: psychology [16] and symptoms [17], with study populations mostly comprising patients with depression, cancer, etc. However, there is limited research on the help-seeking behavior of patients with chronic diseases in response to their chronic health conditions or symptoms, and such studies often lack theoretical guidance. Furthermore, capability, opportunity, and motivation are core factors that influence health help-seeking behavior in patients with chronic diseases. A qualitative study indicates that the information- and resource-seeking skills of patients with chronic pain influence their help-seeking behaviors and methods. Patients with good skills can obtain effective guidance from healthcare professionals to better manage their pain symptoms [18]. However, research has pointed out that patients with stroke, due to insufficient medical knowledge reserves, find it difficult to make judgments about the onset and progression of the disease or its symptoms, and thus cannot make timely and correct decisions for seeking help, which results in delayed assistance [19]. The accessibility of medical resources affects health help-seeking among patients with chronic diseases. Research indicates that rural, regional, and remote communities have poorer access to healthcare services [20]. Inconvenient transportation and long distances to medical institutions also increase the difficulty of seeking professional medical help for patients with chronic diseases. Distrust of medical services and concerns about economic burdens can also weaken the willingness of patients with chronic diseases to seek help proactively. Some patients may be worried about high medical expenses and thus choose to ignore or endure their symptoms as much as possible [18]. This motivation makes their behavior exhibit a polarized feature of “not treating mild cases and being difficult to treat severe ones”. Therefore, it is necessary and practically significant to explore the specific difficulties and facilitating conditions in the help-seeking process from the perspective of patients’ own experiences.

The Capability, Opportunity, and Motivation-Behavior (COM-B) model, introduced by Michie in 2011 [21], highlights that individual behavior is directly or indirectly shaped by three core factors: capability, opportunity, and motivation. The COM-B model has been widely applied in health behavior research, including fields such as chronic disease management and health promotion [22,23]. The strengths of this model lie in its ability to comprehensively analyze both the facilitative and inhibitory factors influencing behavior. Therefore, this study used the COM-B model to explore the facilitating and hindering factors of help-seeking behaviors that concern health issues among patients with chronic diseases.

## 2. Materials and Methods

### 2.1. Study Design

A qualitative descriptive study was designed to explore the influencing factors of health help-seeking behaviors among patients with chronic diseases. The interview guide was developed based on Levkoff et al. (1999)’s proposed help-seeking model [24] to ensure comprehensive coverage of the process of health help-seeking behavior among patients with chronic diseases. In order to conduct a rigorous analysis and classification of the influencing factors, we adopted a more detailed and behavior-oriented COM-B model as the coding framework. This model places greater emphasis on the dynamic interaction between the environment and individual factors [21], which is more in line with the goal of this study to explore multi-level influencing factors. In addition, this model can overcome the limitations of a single factor in traditional analysis and better reflect the actual situation of patients’ health assistance behaviors.

### 2.2. Recruitment and Participants

The purposive sampling method was used to select patients with chronic diseases who were hospitalized in the Department of Neurology of a tertiary hospital in Zhengzhou City, Henan Province from July to November 2024. Patient inclusion criteria: (1) patients with a clear diagnosis—a diagnosis of a certain chronic disease by a medical institution in accordance with relevant clinical guidelines or standards and recorded in the case—of hypertension, diabetes, coronary heart disease, stroke, hyperlipidemia, or one or more of the five diseases; (2) clear thinking, ability to express their feelings, and willingness to cooperate. Exclusion criteria: (1) patients with cognitive or communication impairments or aggravated conditions who are unable to cooperate to complete the interview.

This study adopted a purposive sampling strategy and selected inpatients with chronic diseases as the research subjects. The reasons are as follows: (1) during hospitalization, patients have time and energy to participate in in-depth interviews; (2) hospitalization events caused by symptom changes or poor management are often critical moments that trigger patients to reflect on their health help-seeking behaviors, providing richer and deeper help-seeking information; (3) most of the patients hospitalized in the neurology department are patients with stroke. They usually have complex experiences of seeking help when it comes to disease management and rehabilitation. The experiences of patients with other chronic diseases (such as respiratory diseases) in different environments (such as primary care, community clinics) may generate different promoting factors and obstacles; (4) it is convenient for researchers to access and recruit diverse samples of patients with chronic disease that meet research criteria in a relatively controllable environment.

### 2.3. Research Framework

Levkoff et al. (1999) proposed [24] a help-seeking model which focuses on the psychological and behavioral changes in the process of seeking help by analyzing them through four stages: disease symptom experience, symptom assessment, decision to seek care, and contact with care providers. This model has been applied to the study of the help-seeking behavior of patients with Alzheimer’s disease to explore the reasons for their poor help-seeking behavior [25]. According to the purpose of this study, we used this model to develop an interview outline to explore the health help-seeking behavior of patients with chronic diseases. Before the formal interview, two patients with chronic diseases were pre-interviewed, and the interview results were not analyzed. Based on the results of the pre-interview, a formal interview outline was formed after discussion by the research group. The outline of the interview is as follows:

Disease and symptom experience

(1) How did you first discover this health issue?

(2) What are your thoughts and feelings about this illness or symptom?

(3) How do other family members or others around you perceive the disease or symptoms? What are your thoughts and feelings?

Symptom appraisal

(4) What are the main reasons and motivations for seeking help when you have health problems?

(5) How much do you and your family know about your health? What kind of health services are provided in your area?

Decision to seek care

(6) Did you make any psychological or behavioral adjustments or have any reactions before deciding to seek help?

(7) What did you think after you made the decision to ask for help? What aspects have you considered?

(8) What factors do you think may promote or hinder behaviors of seeking health help?

Interpersonal communication

(9) What preparations will you make when you decide to ask medical staff, relatives, friends, and neighbors for help?

(10) What are your needs, expectations, or questions regarding follow-up help and treatment?

(11) In addition to the above questions, what else do you want to communicate and express? Do you have any other experiences or feelings about health help-seeking behaviors that you want to supplement and communicate?

### 2.4. Data Collection

Patients who meet the requirements and are interested were initially introduced to this study by the responsible nurse. Researchers SLL and LXC (female) then had face-to-face contact with the patients, elaborately explaining the purpose, content, voluntariness, confidentiality measures, and possible risks and benefits of this study. After obtaining oral and written informed consent, they arranged the time and place for the interview. All semi-structured interviews were conducted by the principal investigators of this study, SLL and LXC. They are master’s degree holders in nursing, are registered nurses, have received qualitative interview training, and have conducted multiple mock interview practices before the start of this study to ensure the consistency of their interview skills. Face-to-face, semi-structured in-depth interviews were used to collect data during the patients’ hospitalization (Supplementary Materials [26]). The interviews were conducted in the office of the department in a quiet environment without disturbances from other people. No one else was involved except the patient and the interviewer. Each interview lasted 30–40 min and each patient was interviewed once. No repeated interviews were conducted. The quality of the interview was ensured through the following measures: (1) The interviewer established a relationship of trust with the respondents before the interview so that they could fully express their inner thoughts. (2) In-depth interviews were conducted according to the interview outline, and the order of questions was adjusted as appropriate to ensure that they did not deviate from the topic. During the interview, patients were encouraged to fully express their thoughts and feelings about health help-seeking behavior, listen carefully, clarify in time, ask questions in a timely manner, and avoid guidance and suggestive language. (3) A voice recorder was used during the interview. Notes were taken on the spot to record the interviewee’s facial expressions and body movements and the meaningful words repeatedly mentioned by the interviewee. (4) After the interview, the interview materials were sorted out in a timely manner, without personal preconceptions and biases, and the interviewees were asked to confirm and verify the text materials after they were organized. (5) Numbers were used to represent patient information and organize the interview records by date.

### 2.5. Data Analysis

The interview recordings were conducted on-site with a voice recorder and transcribed verbatim using “iFlytek Hearing 3.0.” software. The accuracy of the transcribed text was verified by the interviewers SLL and LXC. The interviews were transcribed by the interviewer within 24 h after the interviews and the transcriptions were supplemented by proofreading in conjunction with field notes. This result was returned to the patient for verification. The analysis process was initially encoded independently by two researchers, YX and WXF. Directed content analysis was used to analyze the data, following a process encompassing three stages: preparation, organization, and reporting [27]. Coding was performed using Microsoft Word. In the preparation stage, the analysis unit was defined as the complete sentence that reflected influencing factors of health help-seeking behaviors of patients with chronic disease. Researchers immersed themselves in the raw data and repeatedly read it to familiarize themselves with the content and context. Following this immersion, a preliminary analytical framework was established with the COM-B model at its core. An initial coding scheme was developed directly from the core dimensions of the COM-B model. Simultaneously, researchers remained open to new information within the data that was not covered by the model and prepared to perform supplementary coding. In the organization stage, systematic coding was implemented. First, data were categorized and labeled according to the initial coding scheme. For data content that could not be classified, supplementary coding was conducted to generate new codes. After all coding was completed, all codes were grouped based on semantic similarity into corresponding categories. Based on this, further inductive summarization and refinement were undertaken to form themes and sub-themes. In the reporting stage, the analysis results were interpreted and synthesized to establish connections between the information and the findings. Representative excerpts were selected from the raw data as supporting evidence. The research team held regular meetings to discuss coding standards, theme extraction, and sub-theme identification. Through comprehensive discussion, consensus was reached, and any discrepancies were resolved. The final thematic framework was reviewed and confirmed by a senior researcher, ZCH (professor).

### 2.6. Rigor and Trustworthiness

This study employed the following methods to enhance its rigor and reliability. First, in this study, the maximum difference method was used to select different participants for interviews and provide detailed descriptions of the respondents’ information. Second, the sample size is based on the principle that no new themes emerged from the interviews and information saturation was reached. In this study, the information of 18 patients was basically saturated after the interviews. No patient withdrew or refused to participate in the interview. Two more patients were interviewed to verify that the interview information reached saturation [28], and no new topics emerged. The interview was concluded. Third, the use of the original words of the interviewees in the research results helps readers make judgments on the accuracy of the analysis results, thereby enhancing the verifiability of the research.

### 2.7. Ethical Considerations

The research protocol and sampling have been approved by the Ethics Committee of Zhengzhou University (ZZUIRB2024-18). Patients signed an informed consent form after being briefed by the investigator on the purpose, methods, and procedures of this study. The patients were informed that the entire interview would be audio-recorded, and they were promised that the audio recordings would be used only for this study.

## 3. Results

### 3.1. Demographics of Participants

This study finally included 18 patients with chronic disease, 9 males and 9 females, who were aged from 51 to 73 years. The one-on-one interview lasted 38–45 min, and there was no second interview. The characteristics of the participants are shown in Table 1 and Table 2.

### 3.2. Thematic Analysis

Based on the COM-B model, the influencing factors of the health help-seeking behavior of patients with chronic diseases are identified and classified. We discovered 2 themes, 6 subthemes and 12 subcategories. Selected quotes taken verbatim from the participants were used to highlight the various themes. The results are shown in Table 3.

#### 3.2.1. Facilitating Factors

##### Sub-Theme 1: Capability

Self-health monitoring capabilities

The interviews found that some patients with chronic diseases were able to quickly identify abnormal physical signals based on the medication experience and disease knowledge that they accumulated in the process of long-term disease management, and gradually formed the ability to self-check symptoms. When uncomfortable symptoms appear, patients will take the initiative to take scientific and effective measures to seek help by analyzing the characteristics and duration of symptoms to avoid aggravation of the disease. P14: “I have this disease (high blood pressure) dizziness lasts for more than 2 h without relief, be careful with high blood pressure, measure the blood pressure at home, if the blood pressure is too high, I will contact the community doctor immediately, never wait, if it is serious, it will be a big trouble.” P8: “In the past few years, I have found my way, such as sudden palpitation, chest tightness, and other small signs, I immediately stopped to rest, although it did not take a few minutes to recover, I still have to find a doctor immediately, otherwise I may have a heart attack. Now I have nitroglycerin and aspirin in a prominent place on my bedside table. Every follow-up visit, I carefully wrote down every indicator that the doctor said, and now that something is slightly wrong with my body, I can tell if it is a blood pressure fluctuation or a new symptom.”

Sufficient communication readiness

The interviews found that some patients with chronic diseases showed an obvious sense of communication readiness in the process of seeking health help, and achieved efficient doctor–patient interaction by sorting out health information in advance, sorting out disease course data, and presetting consultation questions. P13: “I’ve been a little anxious about my recent blood sugar fluctuations, so I’ve compiled a two-week blood sugar monitoring record, including breakfast combinations, exercise times, and medications. Now I still have a habit of circling abnormal data before each visit, and I can communicate with the doctor very clearly.” P17: “The children bought a smart bracelet, which can measure the heart rate and remind me to take medicine, last week’s heart rate was suddenly fast, after the bracelet alarmed, I immediately took pictures and saved them, with the retained data pictures, immediately contacted the community doctor, they gave me timely help.”

##### Sub-Theme 2: Opportunity

Health support in social bonding

The interviews found that the spousal support and good neighborhood interaction of some patients with chronic diseases promoted them taking the initiative to seek help. P14: “After I checked for high blood lipids, my wife would urge me to go out for a walk at night, and our village also organized a walking team, shouting at each other every day to exercise, I have been insisting very well, recently rechecked, triglyceride indicators down, I am happy, and my body is full of energy (referring to the management of high blood lipids).” P3: “Since I got sick, my left hand and left foot did not obey the call, and my wife supports me to practice walking and drags me to rehabilitation every day. She searched the Internet for the rehabilitation experience of stroke patients on her mobile phone and encouraged me to say that if she persists, there is hope for recovery. So I’ve been rehabilitating and now I’m able to walk a few steps on crutches on my own.” At the same time, P11 and P16 said that religious belief plays a certain psychological role in their healthy help-seeking behavior; “In the church, there are people who are willing to listen to my story, I can pour out my inner pain to others without reservation, there are many people in the church, I don’t feel lonely anymore, and every time I go back from the church, my heart is full of strength.”

Effective support from medical staff

The interviews found that the effective support of healthcare workers is an important factor in encouraging patients with chronic diseases to seek help. P18: “The nurse told me about the possible side effects of the hypoglycemic drugs that I’m using now, taught me to determine whether it’s a side effect or a hypoglycemic episode for some of the conditions that occur after the medication is used, and gave me a health education brochure that was produced in the department, and that was quite helpful to me.” P2: “I need to use a grip machine every day for rehabilitation, and the doctors and nurses check my movements again to see if they are standardized, and the doctors also give me a daily training plan, adjust the intensity regularly, and the rehabilitation training is very effective, and I’m much more solid in my heart.”

##### Sub-Theme 3: Motivation

Good illness identity

The interviews found that patients with chronic diseases with good illness identity were self-correcting, actively adjusting, actively coping with various health problems, and seeking help from the outside world promptly. P1: “I know that my illnesses require long-term medication and I’ve accepted them. I often measure my blood sugar and blood pressure, and once there is an abnormality, I will search for health information on the Internet, analyze the cause of the abnormality, and sometimes adjust it myself according to the situation, and sometimes go to the hospital to find a doctor.” P12: “I regard stroke as a challenge in my life, and I don’t miss any possible chance of recovery, incorporating the healthy lifestyle I have learned into my daily life and taking the initiative to change bad habits.” P6: “My family asked me to make a lot of health-friendly changes, and I did them all, such as going for a walk, which made me feel more energetic.”

Past successful experience of health seeking help

The interviews found that some patients with chronic diseases were guided to their follow-up health help-seeking behaviors based on their own effective help-seeking experiences in the past or learning from the successful help-seeking experiences of others. P8: “In the process of seeking help from the doctor, the doctor told me a lot about coronary heart disease and learned a lot of practical knowledge. Now I know how to take care of myself, and I go to the doctor if I have any problems.” P10: “I was suffering from high blood pressure before, and I tried all kinds of home remedies, but my blood pressure was unstable. Later, I went to ask a few patients about their experiences, and they all said that the key was to take antihypertensive drugs. After seeking help from the doctor and taking medicine regularly, my blood pressure has stabilized and my headache has lessened. Therefore, if you encounter any problems, you still have to find someone to ask more.” P2: “I didn’t pay much attention to the rehabilitation training after the operation, but then I participated in the patient symposium, and one of the patients shared his exercise knowledge, and his limb functions have recovered very well and there is no sign at all that he has been ill. I also started to exercise with a grip strength device every day, and I never stopped. Now, my recovery is pretty good.”

#### 3.2.2. Theme 2: Hindering Factors

##### Sub-Theme 1: Capability

Symptom attribution bias

The interviews found that some patients with chronic diseases are affected by symptom attribution bias and often misattribute disease-related abnormal signs to non-pathological factors, which leads to a delay in seeking help for health problems and the continuous progression of the disease in a hidden state. P16: “Before the onset of the disease, I thought it was because I had been sitting for a long time and my blood was blocked, so I just rubbed it. Walking up the stairs and hitting the door frame crookedly, I also told my daughter that I was old and had poor balance, which is normal. As a result, when I woke up the next morning, I found that half of my body couldn’t move and couldn’t speak, so I panicked. After being hospitalized, I learned that numbness in my hands and feet and uncontrollable drooling were all manifestations of cerebral infarction, and I might be able to reduce the sequelae if I came to the hospital early…” P4: “When I was cooking at home at noon, my eyes suddenly turned black, and my right hand couldn’t be used, I thought that low blood sugar was committed, and it would be good to eat, and my family said to go to the hospital to see, I still felt fussed, and now I really regret thinking about it, resulting in slurred speech.”

Difficulty in identifying health information

The interviews found that some patients with chronic diseases face obstacles such as credibility assessment and scientific verification in the screening of massive health information. P1: “There is a flood of diabetes management information, and it can be exhausting to distinguish between what is advertised and what is recommended by a medical professional, let alone verify the effectiveness of these methods. So nowadays, I rarely search for health information on the Internet, and I rely on my own feelings.” P12 said: “There is too much information about diabetes on the Internet (shaking head), there is a video saying that it is good to eat pumpkin, you can control sugar, and insist on eating pumpkin in the morning; Relatives sent a message saying that eating more jealousy lowers blood sugar, and they will put vinegar when cooking. I didn’t know who to believe, and everyone in the patient group also had their own opinions, in the end, I couldn’t even grasp the basic principles of dietary therapy.”

##### Sub-Theme 2: Opportunity

Heavy economic burden

The interviews found that some patients were concerned that the cost of treatment would be too high and beyond their financial means and that they were delaying seeking help as a result. P5: “I usually have some rehabilitation exercises at home, and once I had a follow-up, the neurologist said that my exercise was wrong, and I felt that it would cost me money to go to the rehabilitation department, and I couldn’t earn money now (sigh). I’m very worried and troubled.” P15: “The heart has been uncomfortable for a long time, I heard that coronary angiography is needed for the examination, and if necessary, stents need to be placed. It will cost a lot of money, so wait.”

Poor sense of gain in interactions

The interviews found that for many patients with chronic diseases, a lack of emotional support and unmet needs in interpersonal interactions not only diminished their sense of gain but also hindered their health help-seeking behaviors. P11: “When I talked to my child about the pressure of my recovery, she tried to comfort her but always said ‘There’s nothing we can do about that’, it’s not that I didn’t want to recover, but she didn’t understand my feelings, and it is difficult to give more company and confiding, which made me feel very tired.” P8: “My relatives knew that I had diabetes, so they had to recommend me various home remedies. I explained to them that the medicine prescribed by the doctor was a formal treatment, but they thought I was too rigid and said ‘multi-pronged’, even if it didn’t work, it wouldn’t affect anything, but it made a mess in my mind, and I didn’t want to talk to them about it anymore.”

##### Sub-Theme 3: Motivation

Fear and avoidance

The interviews found that some people with chronic illnesses are apprehensive about possible test results or fear being told that their condition is worse and that this fear may lead to avoidance behaviors that delay seeking help. P7: “Every time the doctor says that I need to check the fundus and urine protein, I panic. Last year, my vision began to deteriorate, and I think it is related to presbyopia, and the doctor warned that diabetes may affect the fundus. I don’t want to recheck—if I don’t check it, it won’t deteriorate, anyway, it won’t affect my life now, I don’t dare to go.” P10: “I didn’t go for a physical examination, I found out a new problem, and I had to take medicine, I took more medicine than food at every meal, how much damage to the liver was, forget it, just maintain the status quo.”

Stigma of illness

The interviews found that some people with chronic diseases have a stigma of illness, which leads to impaired self-esteem and active social avoidance and thus prevents them from seeking health help. P3: “I used to be the breadwinner of the family, but now that I have this disease, I can’t do anything, and I don’t want my old classmates and colleagues to know, let alone take the initiative to ask them for help, it’s too embarrassing.” P9: “I don’t want to go to crowded places, I don’t want others to see that I can’t walk, and I don’t want to participate in health activities organized by the community.”

## 4. Discussion

### 4.1. Strengthen the Factors That Promote the Health of Patients with Chronic Diseases, and Effectively Promote Patients to Actively Seek Help

Based on the COM-B model, this study identified the factors that promote the health help-seeking behavior of patients with chronic diseases, including the patients’ own health monitoring ability, an adequate communication preparation ability, health support in social bonds, effective support from medical staff, good illness identity, and successful experience in previous health help-seeking behavior.

(1) Capability factors: Patients’ health monitoring ability and their communication readiness ability constitute the core competency basis for active help-seeking, which is consistent with previous studies [29,30]. Patients with the ability to identify symptoms, monitor signs, and warn of risks when they encounter health problems can clearly describe their health status, integrate the available health resources around them, and seek appropriate ways to seek help in a timely manner. Symptom identification is a crucial step in self-managing chronic diseases. However, a cross-sectional study pointed out that many patients failed to identify symptoms in time, which delayed the treatment of health problems [31]. A randomized controlled trial pointed out that virtual communities can promote communication among patients and encourage them to actively seek available health resources around them, which effectively enhances patients’ self-management ability [32]. Therefore, medical staff can cultivate patients’ health monitoring and communication skills: on the one hand, they can use the Teach Back technology—a communication method that verifies the patient’s understanding level by having them repeat key health information in their own words—to implement health education [33], improving patients’ health literacy. Medical staff can also develop localized chronic disease communication templates to lower the threshold for patients to express their health demands and improve their communication skills with healthcare professionals. On the other hand, mobile apps or WeChat applets connected to the health language model can be developed to help patients implement health monitoring, early warning, and interpretation of abnormal indicators, so as to achieve professional disease management for patients.

(2) Opportunity factors: The results of this study suggest that health synergy in social bonds and effective support from medical staff are important factors that promote the health help-seeking behavior of patients with chronic diseases, which is consistent with the results of Liu et al. and Ohta [34,35]. The reason for this may be that the encouragement and companionship of family and friends have enhanced the patient’s self-confidence in solving health problems. Additional factors are professional guidance from medical staff, the provision of individualized disease prevention strategies, etc., which enable patients to make decisions to seek help when they encounter health problems. This suggests that a good interactive environment can activate patients’ enthusiasm and initiative to seek help [36], and that medical staff should carry out family health coach programs to train family members to become the backbone of encouraging patients to seek help. A qualitative study on help-seeking behavior pointed out that a close relationship with health professionals, including effective communication, is at the core of obtaining a positive healthcare experience. Perhaps, through intervention measures that provide communication opportunities, patients can improve their ability to seek help and manage pain effectively [37]. In addition, the digital information platform is used to realize the closed-loop management of online consultation and offline follow-up, providing a realistic way for patients to seek help when they encounter health problems.

The results of this study reveal that religious belief has become a source for some participants to obtain social support and psychological comfort, helping them cope with diseases and possibly influencing their opportunities to seek help. This is in line with the growing recognition that spirituality is part of holistic care [38]. Although not all patients with chronic diseases in this study had religious beliefs, this point emphasizes that medical staff need to attach importance to patients’ religious beliefs, make them feel respected and cared for when they are seeking help on health issues, and enable them to address the challenges brought by disease management from the perspective of religious beliefs.

(3) Motivational factors: Good illness identity and successful experience in previous health help-seeking are important promoting factors. The results are consistent with the research results of scholars such as Low and Clur [39,40], who found that after patients adapted to their patient identity, they found the fulcrum of disease management, took the initiative to carry out health management, found health problems, and sought help in time. When patients effectively improve their symptoms through effective help-seeking, a positive mechanism of help-seeking and benefit is formed, and this successful experience can significantly strengthen their belief in the utility of help-seeking and increase their motivation to solve health problems. According to the theory of self-determination, it can be seen that improving patients’ sense of autonomy and competence in seeking help can enhance their motivation to seek help [41]. Therefore, it is suggested that medical staff and patients jointly formulate personalized disease management goals, starting with simple tasks such as recording the patient’s medication status [42] and then introducing more complex self-management tasks after the patient’s ability is improved to strengthen their motivation for help

The 2030 Agenda for Sustainable Development emphasizes enhancing the health level of the entire population, improving the accessibility of medical services, and promoting health awareness [43]. The results of this study show that patients with chronic diseases can obtain help from medical professionals through active health help-seeking behavior, thereby mitigating health inequality caused by the uneven distribution of medical resources, while also improving their health literacy to achieve better self-management and delaying the progression of diseases.

### 4.2. Focus on the Barriers to Health Help-Seeking Behaviors of Patients with Chronic Diseases, and Actively Formulate Strategies to Improve Health Help-Seeking Skills

This study found that the barriers to health help-seeking behavior in patients with chronic diseases included symptom attribution bias, difficulty in identifying health information, a heavy economic burden, a poor sense of gain in interaction, avoidance and fear, and stigma.

(1) Capability factors: Symptom attribution bias and difficulty in identifying health information are important obstacles to seeking help, which is consistent with the results of Shtompel and Gallagher [44,45]. These obstacles may be related to the lack of disease knowledge in patients with chronic diseases. In addition, the patients’ help-seeking behavior is not static, but a dynamic process that is constantly adjusted as the disease progresses [46], so it is necessary to continuously strengthen their understanding of the disease. Therefore, it is suggested that medical staff should strengthen the education of patients during the critical window period for seeking help. One example of this is simulating real scenarios such as disease fluctuations, drug reactions, and sudden symptoms using the scenario analysis method [46]. Another is constructing an internet knowledge dissemination matrix to empower patients to transform fragmented health knowledge into health coping strategies to improve their disease cognition. The study of Low [47] pointed out that actively obtaining health information related to disease management can enhance the health help-seeking awareness and self-management behavior of patients with chronic diseases, which suggests that medical staff can develop a hierarchical and classified health information database, and label and organize the content according to the type of disease, urgency, etc., so as to allow users to quickly obtain health information. In this health information database, the search engine should introduce AI credibility scoring to score the source of health information. Social platforms such as WeChat and Douyin set up health information alerts to trigger risk warnings for unverified medical content.

(2) Opportunity factors, such as a poor sense of access to interaction and a heavier economic burden, greatly hinder patients with chronic diseases from actively seeking help, which is consistent with the findings of Loraine and Liu. [19,48]. On the one hand, chronic disease management requires long-term investment. On the other hand, there is an unmet need in interpersonal interactions. These research findings support healthcare professionals in promoting easily accessible and low-cost intervention measures from the perspectives of capacity, opportunity, and motivation, thereby improving patients’ health help-seeking behaviors. Healthcare professionals have incorporated low-cost health guidance into routine care. By holding monthly health dialogues in rural areas to share knowledge and experience, it is possible to carry them out among the group of patients with chronic disease with heavy economic burdens and enhance their awareness of their own diseases [35]. In addition, healthcare professionals proactively ask patients during follow-up visits whether their previous health assistance needs have been met and provide targeted feedback. Some research indicates that, in rural Japan, integrating family doctors into various community interactions to provide effective healthcare services can promote effective help-seeking behavior and enhance the quality of life and self-efficacy in self-management of patients with chronic diseases [49].

In recent years, virtual simulation technology has provided an innovative way to reduce economic expenditure and enhance interactive benefits [50], and immersive medical interaction scenarios have a promoting effect on the health management of patients with chronic diseases [51]. For patients, an immersive health help-seeking behavior training system can be designed, integrating VR disease scene simulation and AI real-time feedback functions, to provide real-time behavior modification suggestions, guide patients in learning by trial and error in a safe environment, and provide digital solutions for optimizing the quality of interaction. With the development of technology, this is a foreseeable scenario in the near future. However, we must admit that the large-scale application of this technology still awaits long-term development, and its popularization will require policy and infrastructure support [52].

Therefore, medical staff should take into account the preferences of patients, as well as provide valuable guidance on health help-seeking, in order to promote patients seeking health assistance.

(3) Motivational factors: avoidance, fear, and stigma of illness hinder the help-seeking behavior of patients with chronic diseases, which is consistent with previous studies [37,53]. When patients regard chronic disease management as a controllable part of life rather than a fatal threat, the stigma is significantly reduced, and the willingness of patients to seek help can be enhanced. Research indicates that helping patients and healthcare professionals enhance their communication skills in healthcare and attempting to eliminate the stigma of sensitive and potentially embarrassing symptoms could be the key to increasing patients’ willingness to seek help proactively [54]. Therefore, it is suggested that medical staff introduce digital storytelling: use voice diaries, micro-videos, and other forms to record the turning points of help-seeking and encourage patients to share their mental journey regarding healthy help-seeking behaviors; organize patient discussion meetings to exchange health management experience and stimulate self-management confidence.

### 4.3. Limitations and Future Prospects

This study may have some limitations and only focuses on patients with chronic diseases in the neurology department of a tertiary hospital in Zhengzhou. Patients with chronic diseases who are hospitalized in the neurology department due to symptom changes or poor management have more complex experiences of seeking help, providing richer and more in-depth information regarding health help-seeking behaviors. However, in rural grassroots areas or communities, due to different environments, the convenience, obstacles, and satisfaction with seeking help perceived by patients with chronic diseases may vary. Therefore, in the future, attention can be paid to patients with chronic disease who are in communities and rural areas to enrich the research results on health help-seeking behaviors. In addition, longitudinal qualitative research can be carried out on patients with chronic diseases with different diseases, disease courses, or complex comorbidities to explore the deep causes that affect the health-related help-seeking behaviors of patients with chronic diseases of different diseases and stages, and to deeply deconstruct their dynamic evolution mechanisms.

## 5. Conclusions

Based on the COM-B model, this study constructed an interview outline based on the help-seeking model and explored the promotion and obstacle factors of health help-seeking behavior of patients with chronic diseases, from which two themes and six sub-themes were extracted:

Facilitating factors:

- Capability factors: self-health monitoring ability, sufficient communication preparation ability;
- Opportunity factors: health support in social bonds, effective support of medical staff;
- Motivational factors: good illness identity, past successful experience of seeking health help.

Hindering factors:

- Capability factors: symptom attribution bias, difficulty in identifying health information;
- Opportunity factors: heavier financial burden, poor sense of gain in interactions;
- Motivational factors: fear and avoidance, stigma of illness.

The research results show that encouraging patients with chronic diseases to actively seek help is a crucial part of their long-term chronic disease management:

- Patients with chronic diseases who actively seek help for their health problems may improve their quality of life and slow down the progression of the disease;
- Furthermore, in combination with the COM-B model, future healthcare professionals can provide personalized guidance for patients’ help-seeking behaviors;
- More importantly, patients with chronic diseases, especially those in rural areas, taking the initiative to seek help in chronic disease management is also conducive to the prevention and control of chronic diseases at the grassroots level and promotes health equity.

## Figures and Tables

**Table 1:** Socio-demographic characteristics of patients with chronic disease (N = 18).

Number	Gender	Age	Location	Marital Status	Career	Religion	Education
P1	Female	55	Urban	Married	Accountant	Buddhism	Junior college
P2	Male	66	Urban	Divorced	Worker	None	Junior college
P3	Male	51	Rural	Married	Unemployed	None	High school
P4	Female	54	Urban	Married	Conductor	None	Junior college
P5	Male	61	Rural	Married	Worker	None	Junior high school
P6	Female	67	Rural	Married	Unemployed	None	Primary school
P7	Female	69	Urban	Married	Worker	None	Primary school
P8	Male	70	Urban	Married	Civil servant	None	High school
P9	Female	56	Urban	Married	Retired	None	Junior college
P10	Male	59	Rural	Married	Worker	None	Junior high school
P11	Female	53	Rural	Married	Farmer	Christianity	Primary school
P12	Female	71	Urban	Married	Accountant	None	Junior high school
P13	Male	62	Urban	Divorced	Worker	None	High school
P14	Female	67	Rural	Spouse Deceased	Worker	None	Junior high school
P15	Male	64	Urban	Married	Technicist	None	Technical secondary school
P16	Male	65	Rural	Married	Farmer	Christianity	Junior high school
P17	Male	73	Urban	Married	Teacher	None	Technical secondary school
P18	Female	58	Urban	Married	Worker	None	High school

**Table 2:** Clinical characteristics of patients with chronic disease (N = 18).

Number	Disease
P1	Stroke Diabetes
P2	Stroke, High blood pressure
P3	Stroke, Diabetes
P4	Stroke
P5	Stroke, Coronary heart disease
P6	Stroke
P7	High blood pressure, Diabetes
P8	Coronary heart disease, Diabetes
P9	Stroke
P10	Stroke, High blood pressure
P11	Stroke
P12	Stroke, Diabetes
P13	Diabetes
P14	High blood pressure, Hyperlipidemia
P15	Coronary heart disease
P16	Stroke
P17	Coronary heart disease, Diabetes
P18	High blood pressure, Diabetes

**Table 3:** Classification of themes, subthemes, and subcategories.

Theme	Subthemes	Subcategories	Representative Quotation
Facilitating factors	Capability	Self-health monitoring capabilities	measure the blood pressure at home, if the blood pressure is too high, I will contact the community doctor immediately, never wait. (P14)
		Sufficient communication readiness	I’ve compiled a two-week blood sugar monitoring record, including breakfast combinations, exercise times, and medications. (P13)
	Opportunity	Health support in social bonding	my wife supports me to practice walking and drags me to rehabilitation every day. (P3)
		Effective support from medical staff	the doctors and nurses check my movements again to see if they are standardized. (P2)
	Motivation	Good illness identity	I know that my illnesses require long-term medication, but that’s where I am now, and I’ve accepted them. (P1)
		Past successful experience of health seeking help	the doctor told me a lot about coronary heart disease and learned a lot of practical knowledge. Now I know how to take care of myself. (P8)
Hindering factors	Capability	Symptom attribution bias	before the onset of the disease, I thought it was because I had been sitting for a long time and my blood was blocked. (P16)
		Difficulty in identifying health information	I rarely search for health information on the Internet, and I rely on my own feelings (P1)
	Opportunity	Heavy economic burden	It will cost a lot of money, (P5)
		Poor sense of gain in interactions	it’s not that I didn’t want to recover, but she didn’t understand my feelings, which made me feel very tired (P11)
	Motivation	Fear and avoidance	I don’t want to recheck—if I don’t check it, it won’t deteriorate, anyway, it won’t affect my life now, I don’t dare to go. (P7)
		Stigma of illness	I don’t want others to see that I can’t walk, and I don’t want to participate in health activities organized by the community. (P9)

## Data Availability

The original contributions presented in this study are included in the article/Supplementary Material. Further inquiries can be directed to the corresponding author(s).

## Supplementary Materials

The following supporting information can be downloaded at: https://www.mdpi.com/article/10.3390/healthcare13172164/s1, File S1: COREQ (COnsolidated criteria for REporting Qualitative research) Checklist. Reference [26] is included in the Supplementary Materials.
